# Prevalence and Patient-Level Correlates of Intentional Non-Adherence to Immunosuppressive Medication After Heart-Transplantation—Findings From the International BRIGHT Study

**DOI:** 10.3389/ti.2023.11308

**Published:** 2023-07-10

**Authors:** Mark T. Marston, Lut Berben, Fabienne Dobbels, Cynthia L. Russell, Sabina de Geest

**Affiliations:** ^1^ Nursing Science, Department of Public Health, University of Basel, Basel, Switzerland; ^2^ Pediatric Intensive Care Unit, University Children’s Hospital Basel, Basel, Switzerland; ^3^ Academic Centre for Nursing and Midwifery, Department of Public Health and Primary Care, KU Leuven, Leuven, Belgium; ^4^ School of Nursing and Health Studies, University of Missouri-Kansas City, Kansas City, MO, United States

**Keywords:** immunosuppression, heart transplantation, medication non-adherence, intentional non-adherence, correlates

## Abstract

After heart transplantation (HTx), non-adherence to immunosuppressants (IS) is associated with poor outcomes; however, intentional non-adherence (INA) is poorly understood regarding its international variability in prevalence, contributing factors and impact on outcomes. We investigated (1) the prevalence and international variability of INA, (2) patient-level correlates of INA, and (3) relation of INA with clinical outcomes. Secondary analysis of data from the BRIGHT study—an international multi-center, cross-sectional survey examining multi-level factors of adherence in 1,397 adult HTx recipients. INA during the implementation phase, i.e., drug holiday and dose alteration, was measured using the Basel Assessment of Adherence to Immunosuppressive Medications Scale^©^ (BAASIS^©^). Descriptive and inferential analysis was performed with data retrieved through patient interview, patient self-report and in clinical records. INA prevalence was 3.3% (*n* = 46/1,397)—drug holidays: 1.7% (*n* = 24); dose alteration: 1.4% (*n* = 20); both: 0.1% (*n* = 2). University-level education (OR = 2.46, CI = 1.04–5.83), insurance not covering IS costs (OR = 2.21, CI = 1.01–4.87) and barriers (OR = 4.90, CI = 2.73–8.80) were significantly associated with INA; however, clinical outcomes were not. Compared to other single-center studies, this sample’s INA prevalence was low. More than accessibility or financial concerns, our analyses identified patient-level barriers as INA drivers. Addressing patients’ IS-related barriers, should decrease INA.

## Graphical Abstract

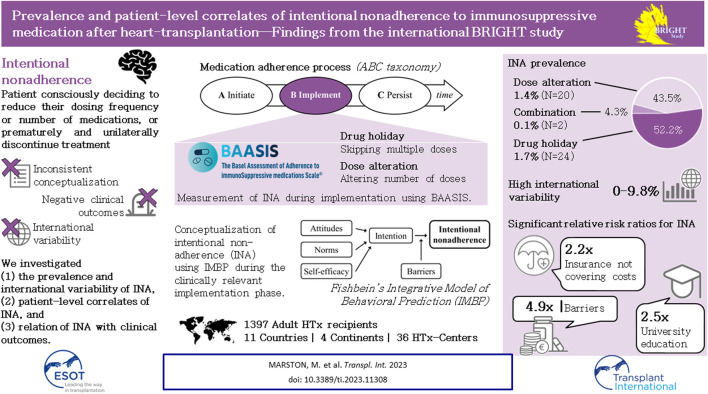

## Introduction

After heart transplantation (HTx), patients need to adhere to a life-long immunosuppressive medication (IS) regimen [1]. Poor adherence to IS has been linked to poor clinical and economic outcomes [2].

**FIGURE 1:**
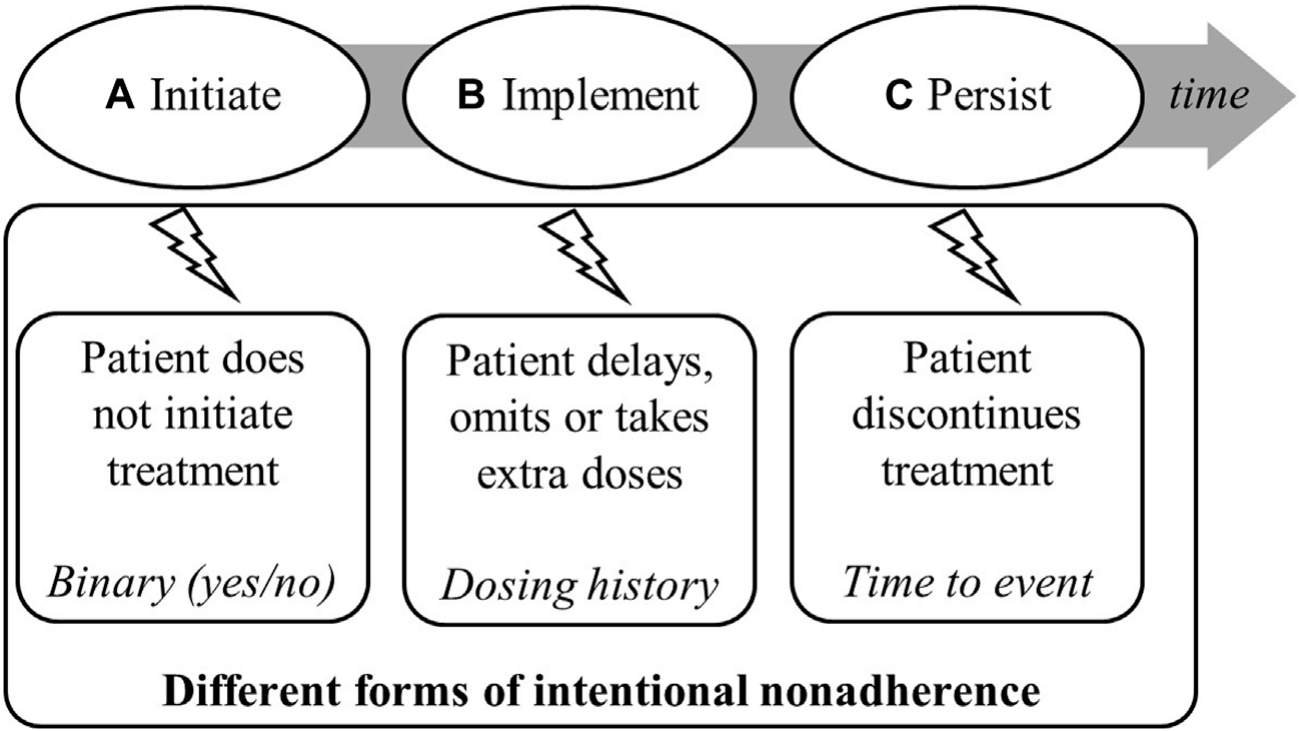
Process of medication adherence illustrating phases in which intentional non-adherence and intentional implementation non-adherence (i.e., drug holiday and dose alteration) may appear [3].

Following the Ascertaining Barriers to Compliance (ABC) taxonomy definition, medication adherence is the process by which a patient follows a medication regimen as prescribed. It has 3 phases: initiation, implementation, and persistence (Figure 1) [3]. While non-adherence can occur during any of these phases, after HTx, initiation of IS takes place under clinical supervision and therefore medication non-adherence (NA) is most common during the implementation and persistence phases [3]. Medication NA can be discerned as either intentional or unintentional [4, 5]. Intentional non-adherence (INA) refers to a rational decision-making process and the ability of a person to act on a behavior [6, 7]. This is opposed to unintentional non-adherence, a passive and intermittent process that results from forgetfulness, a lack of capacity, skills, and/or resources [6–11].

Rational decision-making is related to the ability to formulate and carry out a behavior. Within the context of INA, patients decide to reduce their dosing frequency or number of medications, or even to prematurely and unilaterally discontinue treatment (i.e., non-persistence) [9, 12]. This also includes consciously deciding to skip several consecutive doses (i.e., a drug holiday) or to alter the dose of medication (i.e., dose alteration) [13, 14]. The objective is often to avoid disturbing side-effects, to circumvent a restrictive schedule or taking constraints (e.g., having to take food simultaneously), or to generate a feeling of control [9]. Doses may also be omitted or reduced to make a prescription last longer [15].

To date, though, INA to IS (which we will refer to simply as INA) has received only limited attention in the HTx populations and has not been well-substantiated due to inconsistent definition and measurement and large international variability. INA has not been directly studied, and estimated prevalence of drug holidays or non-persistence to IS vary widely, respectively 0%–7.1% and 0.6%–3.1% [16–18].

Deviations from prescribed medication regimen may adversely influence its effect and put the patient at risk of negative clinical outcomes—acute rejection episodes, graft loss, and death [19, 20]. It is unclear how INA influences this risk and how prevalent it is [2, 21, 22].

The limited evidence on correlates of INA focuses on patient-level barriers: beliefs [11, 23], disruption of daily routine [23, 24], and knowledge gaps [5, 25, 26]. System-level correlates: financial barriers related to a lack of health insurance coverage or other sources of increased out-of-pocket monthly expenses [27–29], vary between healthcare systems and show high international variability in relation to INA.

The aims of this study were to 1) assess the prevalence and variability of INA in adult HTx internationally, 2) investigate patient-level correlates of INA, and 3) assess INA’s associations with clinical outcomes in adult HTx recipients.

## Patients and Methods

### Design and Sample

This is a secondary data analysis of the “Building research initiative group: chronic illness management and adherence in transplantation” (BRIGHT) study [30], an international, multi-center, cross-sectional survey examining multi-level factors related to IS adherence in HTx recipients. Detailed information on the BRIGHT study has been reported elsewhere [27, 30]. In a multi-stage sampling approach, a convenience sample including 11 countries, 36 HTx centers, and a random sample of HTx recipients was selected. Transplant recipients were included using seven criteria [30]: 1) ≥18 years old at time of inclusion; 2) transplanted and followed-up for routine care in participating centers; 3) first transplant; 4) single-organ transplant; 5) 1–5 years post-transplant; 6) could read in the languages spoken in the country of the participating center; and 7) could provide written informed consent. Exclusion criteria were: 1) had participated in an adherence intervention study within the past 6 months; or 2) were receiving professional support in taking medication at the time of this study.

### Variables and Measurement

We based our analyses on data collected using the BRIGHT questionnaires (i.e., BRIGHT patient interview, BRIGHT patient self-report questionnaire) and on the BRIGHT data—including those relating to patient outcomes—collected from clinical files [27, 30]. Intentional NA—drug holidays and dose alterations—patient-level correlates and center location were assessed through patient interview transcripts and patients’ written self-reports [30].

#### Socio-Demographic Data

The following demographic data were assessed (see Table 1 for answer options) [30]: age (in years), gender, marital status, living situation, employment status, educational level (using a standardized categorization across countries), ethnicity and center location/country.

**TABLE 1:** Socio-demographic characteristics and clinical outcomes for total group and patients showing intentional non-adherence.

Variables	Values/scoring	Total sample	Intentional non-adherence	
		N; mean ± SD | N (%)	N; mean ± SD | N (%)	OR^a^ (95% CI)
		N = 1,397	N = 46	
Socio-demographic characteristics				
Age	Years	1,363; 53.7 ± 13.2	45; 49.8 ± 14.3	0.98* (0.96–0.99)
	Missing	34 (2.4%)	1 (2.2%)	
Gender	Female	379 (27.1%)	13 (28.3)	1.05 (0.55–2.02)
	Missing	7 (0.5%)	0	
Ethnicity	Caucasian	1,186 (84.9%)	35 (76.1%)	Reference
	Afro-American	80 (5.7%)	3 (6.5%)	1.27 (0.38–4.23)
	Asian	27 (1.9%)	3 (6.5%)	4.10* (1.17–14.19)
	Hispanic	29 (2.1%)	0	n.a.
	North-African	28 (2.0%)	1 (2.2%)	1.25 (0.17–9.51)
	Other	31 (2.2%)	4 (8.7%)	5.02** (1.66–15.16)
	Missing	16 (1.1%)	0	
Marital status	Married/living with partner	955 (68.4%)	26 (56.5%)	Reference
	Single	242 (17.3%)	11 (23.9%)	1.71 (0.83–3.52)
	Separated/divorced	149 (10.7%)	6 (13.0%)	1.52 (0.62–3.77)
	Widowed	41 (2.9%)	3 (6.5%)	2.81 (0.81–9.68)
	Missing	10 (0.7%)	0	
Living alone	Yes	265 (19.0%)	7 (15.2%)	1.32 (0.58–2.99)
	Missing	15 (1.1%)	0	
Education	< Secondary	370 (26.5%)	8 (17.4%)	Reference
	Completed secondary	328 (23.5%)	9 (19.6%)	1.27 (0.48–3.32)
	Further education	382 (27.3%)	13 (28.3%)	1.58 (0.65–3.85)
	University	308 (22.0%)	16 (34.8%)	2.46* (1.04–5.83)
	Missing	9 (0.6%)	0	
Employment	(Self-)employed (1)	366 (26.2%)	11 (23.9%)	Reference
	Looking for job 2)	40 (2.9%)	3 (6.5%)	2.58 (0.69–9.69)
	(Temp.) unable (5)	404 (28.9%)	18 (39.1%)	1.50 (0.70–3.22)
	Retired (4)	466 (33.4%)	12 (26.1%)	0.85 (0.37–1.94)
	Other (3)	97 (6.9%)	1 (2.2%)	0.34 (0.04–2.63)
	Missing	24 (1.7%)	1 (2.2%)	
Clinical outcomes				
Time since Tx	Years	1,378; 3.4 ± 1.4	45; 3.1 ± 1.4	0.84 (0.67–1.05)
	Missing	19 (1.4%)	1 (2.2%)	
Treated rejections in follow-up	N events per year	1,391; 0.9 ± 1.5	45; 1.2 ± 1.7	1.16 (0.99–1.37)
	Missing	6 (0.4%)	1 (2.2%)	
	No event^b^	840 (60.1%)	23 (50.0%)	Reference
	≥1 event	551 (39.4%)	22 (47.8%)	1.48 (0.82–2.68)

OR, odds ratio; CI, confidence interval. *p < 0.05, **p < 0.01, ***p < 0.001.

^a^ Logistic regression for bivariate analysis [31], bold when significant.

^b^ Dichotomisation, comparison of patients with no treated rejections and patients with one or more treated rejections for bivariate analysis [31].

#### Intentional Non-Adherence

Intentional NA was assessed using 2 items from the 5-item Basel Assessment of Adherence to immunoSuppressive medIcation Scale (BAASIS^©^ https://baasis.nursing.unibas.ch/) [32]. The first item, *drug holiday*, was operationalized for patients indicating they had skipped two or more consecutive doses of medication. The second, *dose alteration*, was operationalized for patients indicating that they had altered their prescribed IS dosage (i.e., they had taken more or fewer pills per dose than prescribed) over the last 4 weeks [27]. Intentional NA was operationalized as a positive answer to either of these two items.

#### IMBP Correlates of Intentional Non-Adherence

**FIGURE 2:**
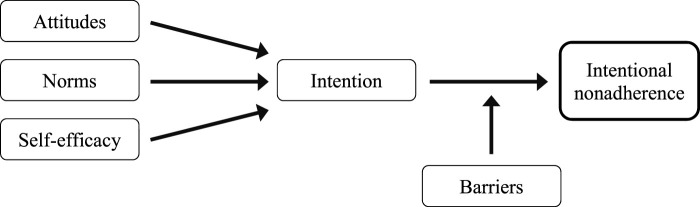
Modified integrative model of behavioral prediction [34, 35].

Fishbein’s Integrative Model of Behavioral Prediction (IMBP; Figure 2) [33] posits that *Intention to perform* is the most proximal determinant of health behavior. Intention to perform has three determinants: *attitudes*, *norms* and *self-efficacy*. An attitude is defined as a positive or negative feeling towards performing the behavior [34]. Subjective norms are defined as the beliefs an individual or a group has regarding whether or not to perform a given behavior [34]. Self-efficacy refers to the person’s beliefs regarding performing a recommended behavior, despite circumstances or barriers making it difficult [34]. Fishbein’s model acknowledged that the presence of personal or environmental barriers may hinder patients from acting upon their intentions and keep them from executing the recommended behavior (Figure 2) [34]. The next paragraphs describe the instruments to measure these five concepts. Information on the instruments’ psychometric properties can be found elsewhere [27].

##### Intention

Intention was operationalized as the cognitive representation of a person’s readiness to perform a given behavior [27]. As an indicator of the capacity of a person to take actions necessary to attain a target [36], it was assessed using 3 investigator-developed items (e.g., “I always intend to take my IS on time”) rated on a unidimensional 5-point Likert-type scale ranging from 1 (strongly disagree) to 5 (strongly agree) [27]. Intention was scored by calculating a mean across the 3 items. This subscale’s Cronbach’s *alpha* was 0.81 [27].

##### Attitudes

Attitudes were operationalized to reflect how favorably—such as important to avoid organ rejection—or unfavorably—such as poison—each patient considered IS. Attitudes are related to a patient’s degree of belief that a given behavior will lead to a favorable or unfavorable outcome [36]. Attitudes were assessed using a 21-item investigator-developed instrument asking patients’ to rate their concerns/worries (12 items, e.g., “Immunosuppressive medications are addictive”) as well as how necessary they considered their IS (9 items, e.g., “Immunosuppressive medications protect my heart”) [27, 30, 35]. Items were rated on a 5-point Likert-type scale ranging from 1 (strongly disagree) to 5 (strongly agree). Total scores for the *positive attitudes*—favorable—and *worries*—unfavorable—dimensions were calculated as the mean score over each item’s rating. The dimensions’ Cronbach’s *alphas* were, respectively 0.77 and 0.66 [27].

##### Norms

Regarding norms, the operational definition used here relates to patients’ perceptions of social pressure or relevant others’ beliefs that may influence their decision-making about medication taking [27]. Important influences may include others’ approval or disapproval of a behavior or the knowledge that some behaviors cannot be performed without assistance [36]. An 11-item investigator-developed instrument based on previous work [24, 37–41] was used to measure normative beliefs about IS (e.g., “Some of my family members disapprove that I have to take immunosuppressive medications”) [24, 27, 30]. Patients were asked to rate items on a 5-point Likert-type scale ranging from 1 (strongly disagree) to 5 (strongly agree). As psychometric analysis confirmed the instrument’s unidimensionality, a mean score was calculated across all items. This instrument’s Cronbach’s *alpha* was 0.94 [27].

##### Self-Efficacy

Self-efficacy was defined as the patients’ confidence in their ability to take their IS in a given situation [27]. This confidence depends on perceived skills and possibly the expected cooperation of others [36]. Regarding IS, self-efficacy behavior was assessed using the 23-item Long-Term Medication Behavior Self-Efficacy Scale [42]. Items were rated on a 5-point Likert-type scale ranging from 1 (not at all confident) to 5 (totally confident). As psychometric analysis showed that this scale is unidimensional, an overall mean score was calculated for self-efficacy. Cronbach’s alpha was 0.98 [42].

##### Barriers

Barriers were operationalized as personal circumstances or environmental constraints that might either prevent a patient from enacting an intended behavior or limit their capacity to perform desired actions [12]. The 19-item IS Medication Adherence Barriers instrument represents barriers identified by patients attempting to follow IS regimens [30]. Items (e.g., “I find it hard to swallow my IS medication”, “I find it hard to take my IS medication because I experience side-effects,” or “I find it hard to go away from home and plan the day because I have to take my IS medication”) are rated on a unidimensional 5-point Likert scale ranging from 1 (never) to 5 (always). A mean score across the 19 items is then calculated. This instrument was developed by the Transplant360 Task Force [43]. Its Cronbach’s alpha was 0.89.

##### Financial Barriers

Financial barriers—healthcare system-level factors—, are cost-related difficulties that hinder a patient from enacting a behavior [36]. Those affecting IS taking are often related to health insurance not or only partially covering the medication costs, necessitating high monthly expenditures [15]. Financial barriers were assessed using six investigator-developed items, which were dichotomized for the purpose of this study: Health insurance covering costs of IS (no versus yes partly, yes fully); Out-of-pocket monthly cost of IS (0–$20, $20.01–$60, $60.01–$110 versus >110$); Feeling that one has enough money to pay for IS (not enough versus mostly enough, enough, more than enough); Prescription for IS not filled because it was too expensive (never versus once, twice, 3–4x, 5–6x, ≥7x); Skipping a dose to make prescription for IS last longer due to lack of money (no never versus yes sometimes, yes often); and Reducing dose to make prescription for IS last longer due to lack of money (no never versus yes sometimes, yes often).

#### Clinical Outcomes

Two clinical outcomes were assessed (see Table 1): time since transplantation (in years); and number of treated rejections experienced per year in follow-up.

### Data Collection

The BRIGHT study’s data collection has been described previously [27, 30]. Data were collected from early 2012–early 2017 [27].

### Data Analysis

We used descriptive statistics as appropriate based on measurement levels and data distributions. Hierarchical inferential statistics, i.e., multilevel logistic regression analysis, was used to assess associations between INA (i.e., drug holiday and dose alteration), IMBP correlates (Figure 2) and clinical outcomes, while controlling for international variability. Socio-demographic characteristics, financial barriers and clinical outcomes that initial analyses suggested were significantly associated with INA were included in the model. Financial barrier-related data were dichotomized before inclusion. Generalized linear regression with random effects was used in the multilevel analysis of international variability. However, the small INA sample size did not allow for moderator analysis with significant or otherwise meaningful results.

Missing data analysis was performed, including a visual analysis with *Amelia II* [44] (multiple imputation software). Analysis of distribution did not reveal any substantial differences between the 20 patients (1.4%) who provided insufficient information relative to BAASIS^©^ to assess adherence [32]. For further analysis, the authors proceeded with list-wise deletion.

The software package used for statistical analysis was R, version 4.0.2, 2020-06-22. [45] Statistical significance was set at *p*<.05.

## Results

### Sample Characteristics

This analysis included 1,397 patients (details provided elsewhere) [27]. Participants’ mean age was 53.7 (±13.2) years; 27.1% were female; 84.9% were of Caucasian origin. At time of interview, most (68.4%) were married or living with partners; 19.0% were living alone. The majority (72.8%) had completed secondary school or higher, with 22.0% holding University degrees; 26.2% were employed or self-employed; 28.9% were temporarily or fully unable to work; and 33.4% were retired. Financial barriers such as health insurance not covering IS costs and high monthly out-of-pocket IS expenses were reported respectively by 9.2% and 9.5% of patients. A more detailed overview of patient-level characteristics can be found in the Tables 1, 2.

**TABLE 2:** Financial barriers for total group and patients showing intentional non-adherence.

Variables	Values/scoring	Total sample	Intentional non-adherence	
		N (%)	N (%)	OR^a^ (95% CI)
		N = 1,397	N = 46		
Health insurance covering costs of IS medication	**No** ^**b**^	128 (9.2%)	8 (17.4%)	2.21* (1.01–4.87)
	Yes, partly	502 (35.9%)	17 (37.0%)	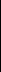 Reference
	Yes, fully	743 (53.2%)	19 (41.3%)	
	Missing	24 (1.7%)	2 (4.3%)		
Out-of-pocket monthly cost of IS medication	**>110$** ^**b**^ ^,^ ^**c**^	133 (9.5%)	8 (17.4%)	2.04 (0.93–4.48)
	60.01–110$	129 (9.2%)	5 (10.9%)	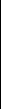 Reference
	20.01–60$	241 (17.3%)	7 (15.2%)	
	0–20$	850 (60.8%)	25 (54.3%)	
	Missing	44 (3.1%)	1 (2.2%)		
Feeling having enough money to pay for IS medication	**Not enough** ^**b**^	243 (17.4%)	9 (19.6%)	1.27 (0.60–2.70)
	Mostly enough	244 (17.5%)	8 (17.4%)	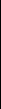 Reference
	Enough	615 (44.0%)	21 (45.7%)	
	More than enough	222 (15.9%)	3 (6.5%)	
	Missing	73 (5.2%)	5 (10.9%)		
Prescription for IS medication not filled because it was too expensive	**Never** ^**b**^	1,349 (96.6%)	44 (95.7%)	1.54 (0.20–11.80)
	Once	7 (0.5%)	0	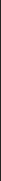 Reference
	Twice	8 (0.6%)	0	
	3–4x	4 (0.3%)	1 (2.2%)	
	5–6x	0	0	
	≥7x	1 (0.1%)	0	
	Missing	28 (2.0%)	1 (2.2%)		
Skipping a dose to make prescription for IS medication last longer due to lack of money	**No, never** ^**b**^	1,344 (96.2%)	44 (95.7%)	1.08 (0.14–8.15)
	Yes, sometimes	21 (1.5%)	1 (2.2%)	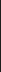 Reference
	Yes, often	8 (0.6%)	0	
	Missing	24 (1.7%)	1 (2.2%)		
Reducing dose to make prescription for IS medication last longer due to lack of money	**No, never** ^**b**^	1,349 (96.6%)	43 (93.5%)	3.16 (0.71–14.01)
	Yes, sometimes	17 (1.2%)	2 (4.3%)	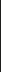 Reference
	Yes, often	4 (0.3%)	0	
	Missing	27 (1.9%)	1 (2.2%)		

OR, odds ratio; CI, confidence interval; IS, immunosuppressive. *p < 0.05, **p < 0.01, ***p < 0.001.

^a^ Logistic regression for bivariate analysis [31], bold when significant.

^b^ Dichotomisation, comparison of bold value against others for bivariate analysis [31].

^c^ According to currency, the cut-off values were set as follows, US$ = 20, 60, 110/£ = 13, 29, 53/€ = 15, 45, 83/CA$ = 19, 57, 104.

### Intentional Non-Adherence

#### Prevalence

Intentional NA was observed in 46 of 1,397 patients (3.3%). Drug holidays were reported by 24 (1.7%), dose alteration by 20 (1.4%). Two (0.1%) reported a combination of drug holiday and dose alteration.

#### International Variability

**FIGURE 3:**
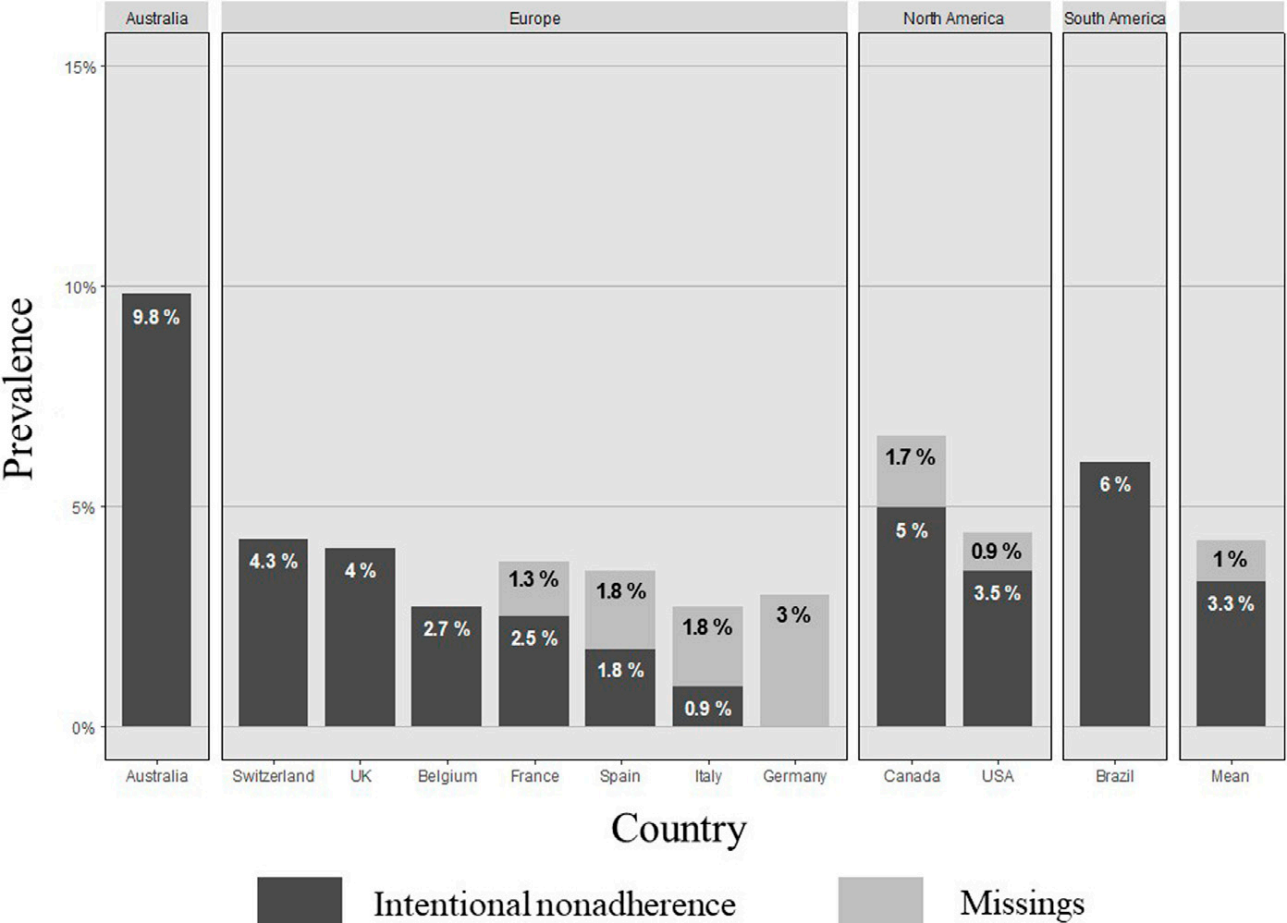
Prevalence of intentional non-adherence internationally. Sample, N (%): Australia, 51 (3.7); Switzerland, 47 (3.4); UK, 99 (7.1); Belgium, 74 (5.3); France, 158 (11.5); Spain, 223 (16.2); Italy, 109 (7.9); Germany, 65 (4.8); Canada, 119 (8.7); USA, 337 (24.3); Brazil, 100 (7.2). Missings: France, 1.3%; Spain, 1.8%; Italy, 1.8%; Germany, 3.0%; Canada, 1.7%; USA, 0.9%; Mean, 1.0%.

International variability was high, with INA prevalence spanning from 0% in Germany to 9.8% in Australia (Figure 3). Drug holidays ranged from 0% in Germany to 4.3% in Switzerland, and dose alteration from 0% in Germany to 7.8% in Australia.

### Correlates of Intentional Non-Adherence

In univariable analyses, lower age (OR = 0.98, CI = 0.96–0.99), being of Asian or *other* origin (OR = 4.10, CI = 1.17–14.19 and OR = 5.02, CI = 1.66–15.16), and university education were associated with higher INA (OR = 2.46, CI = 1.04–5.83). Lack of insurance coverage for IS was the only financial barrier significantly related to a higher risk of INA (OR = 2.21, CI = 1.01–4.87). Low intention was strongly related to INA (OR = 0.54, CI = 0.38–0.77). High worries (OR = 1.81, CI = 1.15–2.85), low self-efficacy (OR = 0.59, CI = 0.44–0.80) and high barriers (OR = 4.90, CI = 2.73–8.80) also significantly increased the odds for INA (Table 3).

**TABLE 3:** Correlates of intentional non-adherence and bivariate analysis.

Variables	Values/scoring	Total sample	Intentional non-adherence	
		N; mean ± SD N (%)	N; mean ± SD N (%)	OR^a^ (95% CI)
		N = 1,397	N = 46	
Predictors of the IMPB model				
Intention to adhere to the immunosuppressants regimen	1 (strongly disagree) to 5 (strongly agree)	1,376; 4.69 ± 0.53	45; 4.41 ± 0.68	0.54*** (0.38–0.77)
	Missing	21 (1.50)	1 (2.17)	
Barriers to take immunosuppressants as prescribed	1 (never) to 5 (always)	1,378; 1.19 ± 0.30	45; 1.47 ± 0.54	4.90*** (2.73–8.80)
	Missing	19 (1.36)	1 (2.17)	
Attitudes towards taking immunosuppressants (dimension positive attitudes/looking towards the future)	1 (strongly disagree) to 5 (strongly agree)	1,375; 4.46 ± 0.46	45; 4.46 ± 0.40	0.99 (0.51–1.89)
	Missing	22 (1.57)	1 (2.17)	
Attitudes towards taking immunosuppressants (dimension worries)	1 (strongly disagree) to 5 (strongly agree)	1,370; 1.91 ± 0.57	45; 2.12 ± 0.60	1.81* (1.15–2.85)
	Missing	27 (1.93)	1 (2.17)	
Perceived norms related to immunosuppressants	1 (strongly disagree) to 5 (strongly agree)	1,241; 1.30 ± 0.60	42; 1.43 ± 0.61	1.36 (0.89–2.08)
	Missing	156 (11.17)	4 (8.70)	
Self-efficacy with taking immunosuppressants	1 (not at all confident) to 5 (completely confident)	1,362; 4.36 ± 0.81	45; 3.95 ± 0.89	0.59*** (0.44–0.80)
	Missing	35 (2.51)	1 (2.17)	

OR, odds ratio; CI, confidence interval. *p < 0.05, **p < 0.01, ***p < 0.001.

^a^ Logistic regression for bivariate analysis [31], bold when significant.

The multivariate analysis of demographic correlates showed that having a university degree was significantly related to INA (OR = 2.95, CI = 1.05–8.29). Intentional NA was strongly associated with the IMBP correlate barriers (OR = 4.81, CI = 2.17–10.65) and insurance not covering IS costs (OR = 2.32, CI = 1.02–5.25).

When controlling for differences between countries (as a random effect), being of Asian origin (b = 0.076, *p* = 0.036), being a widow (b = 0.077, *p* = 0.012), not living alone (b = 0.032, *p* = 0.035) and having a university degree (b = 0.035, *p* = 0.035) correlated with a higher risk of INA. Barriers remained the only IMBP that is associated with a higher risk of INA (b = 0.11, *p* < 0.001).

### Clinical Outcomes

On average, patients had been transplanted 3.4 years (±1.4) and had experienced 0.9 (±1.5) treated rejections per year in follow-up. The proportion of patients who had experienced at least one rejection episode during follow-up was not significantly higher in those reporting INA (*n* = 22/46, 47.8%) than in the overall sample (*n* = 551/1,397, 39.4%; OR = 1.48, CI = 0.82–2.68).

## Discussion

To our knowledge, this is the first study to investigate the prevalence and correlates of INA to immunosuppressive medication after HTx internationally. Its major strengths are its international multisite sample and the use of a theoretical model to guide the exploration of correlates of intentional non-adherence [3, 19, 46, 47].

### Intentional Non-Adherence

Our sample’s overall INA rate, 3.3% (*n* = 46/1,397), was lower than those reported in comparable clinical populations [32]. Few studies have been published distinguishing drug holiday and dose alterations of IS after HTx using the BAASIS^©^; [32, 48–51]. BAASIS^©^, as a self-report method, is embedded in the ABC taxonomy, assessing phases of medication adherence and providing bases for operationalization and assessment of INA (i.e., drug holidays or dose alterations) [46, 52, 53]. Respectively, three and four studies have reported higher prevalence either of drug holidays (8.3%–11.0%) [49–51] or of dose alterations (5.6%–12.1%) [48–51]. Skipping multiple doses—drug holiday—represents a higher risk for negative clinical consequences and is especially concerning [17, 40, 54]. Despite similar medication regimens described, i.e., drug type and twice-daily dosing, patients included in these studies had longer times—4.8 and 7.5 years—since transplantation [48, 49]; and non-adherence has been shown, although inconsistently, to increase over time [55–57]. Compared to non-adherence rates for other types of medication (e.g., adjuvant endocrine therapy in breast cancer: 7%–14%; anti-retroviral therapy in HIV: 17.8%; tyrosine kinase inhibitors in chronic myeloid leukemia: 27%), the rates reported for post-HTx INA to immunosuppressants are among the lowest in literature [52, 58–63]. This may be explained by immunosuppressants’ low forgiveness—the need for extremely close adherence to maintain their effects [64–66]—which focusses patients’ attention very closely on their regimens [67–69]. Compared to recipients of other solid organs—such as lung, liver, and kidney—[17, 25, 54, 70] heart recipients’ low INA rates may also reflect the limited therapeutic options available in case of graft rejection, dysfunction or loss [20, 71]. While kidney recipients have the option, for example, of dialysis or renal transplantation from living donors, a heart transplant is usually a one in a life-time gift [51, 72–75].

#### International Variations and Financial Barriers

Our findings show that INA prevalence varies internationally, the highest rates being observed in Australia (9.8%), Brazil (6.0%) and Canada (5.0%). A range of country-level correlates (e.g., insurance coverage, financial barriers, access to medication) have been offered as explanations [76–78]. Measurable moderating variables, such as low insurance coverage for IS in Australia, the USA and Canada [76], or the perceived financial burden of high monthly out-of-pocket expenses in Switzerland [29] may help explain some disparities. Low accessibility, such as greater distance to the transplant center, does not seem to favor INA. [29, 77] When referring to delayed access to a specialist or higher waiting times for appointments, e.g., Canada and oppositely Germany, low accessibility appears to match higher INA rates. [29] This implies that better organized services help compensate low accessibility and prevent INA. [77].

### Correlates of Intentional Non-Adherence

Belonging to an ethnic minority—more specifically, being of Asian or of other origin—increased the odds of INA. This may result from lower levels of support within these populations [79–81] or variations in social desirability across ethnic groups regarding organ transplantation [80]. Social norms may also increase the tendency to underreport INA in favor of other forms of NA, such as forgetfulness [82]. In line with previous research, having a university degree was significantly related to higher rates of INA [71, 83]. It may be assumed that higher-educated persons feel they have the skills to recognize and weigh IS-related benefits and risks [72]. It also strongly suggests that INA does not arise from a lack of understanding [84] or health literacy [58, 70, 85–87]. Instead, it suggests that INA is more closely related to the decision-making process outlined by the theory of planned behavior [88] and how the patient balances the benefits of following the IS regimen against the risks and barriers, e.g., side-effects, taking constraints or disruption of their normal routines [5, 17, 81, 89].

#### IMBP Correlates of Intentional Non-Adherence

Worries (i.e., negative feelings) towards following the IS regimen as prescribed were particularly strongly related to INA. This supports the idea that intentional behavior, even regarding the weighing-out of necessities and concerns, is tipped more by patients’ fears and worries (e.g., “IS medication is toxic for my body” or “doctors place too much trust in IS medication”) than by clinicians’ assurances that IS is necessary and beneficial [25, 75, 88]. Therefore, a slightly heightened sense of worry could greatly increase a patient’s risk of attempting to modulate the IS’ side-effects (e.g., “When I suffer from uncomfortable side effects, it is best if I reduce the dosage of my IS medication a little”) [90] or to increase their compatibility with daily routines (e.g., “Taking IS medication disrupts my daily life”) [5, 88].

Self-efficacy correlated strongly with *lower* rates of INA. Our results show lower levels of self-efficacy in patients indicating INA than in the overall sample (3.95 ± 0.89 vs. 4.36 ± 0.81, *p* < .01). Self-efficacy relates to patients’ beliefs in their ability to affect a situation. It is demonstrated by patients being confident about taking IS in a given situation [27, 91]. Patients experiencing IS constraints may be tempted to cut back on or briefly halt their IS to limit their side-effects, test their effectiveness or increase their sense of control over their disease and its treatment [92]. When such INA behaviors occur, they reflect low self-efficacy, but foster a false sense of control [5]. This, in turn, leads to intentional and fully conscious non-adherence [91, 93].

Despite the intention to adhere to IS regimen, multiple barriers may hinder a patient from performing the necessary behaviors, such as taking multiple pills at once, taking IS whilst busy with other matters, taking them despite side-effects or having to follow an inconvenient schedule. Consequently, barriers were the strongest predictor of INA. Indeed, even when behaviors are intended, certain barriers can prevent patients from enacting them. This tendency supports the hypothesis that regimen-related constraints, especially difficulties taking IS, are more critical than the suspicion that IS is harmful [58].

Recent findings focusing on cost-related medication non-adherence also show that some financial barriers may relate to patient-level factors rather than healthcare system-level factors, i.e., whether “health insurance covers the cost of IS” or “monthly out-of-pocket expenses for IS [are manageable]” [51, 76, 94]. Examples of patient-level factors include attempts to “make prescriptions last longer” or “delay IS medication refills,” and relate closely to how patients prefer to allocate funds [15, 76]. Regarding INA, these results emphasize the importance of addressing financial barriers at the patient level [76].

### Limitations

The reliability of patient self-report is strongly dependent on the data collection techniques used, e.g., patient interview, and on how the patient understands collected information will be used. Both the wording of questions and the interviewer’s attitude may influence the accuracy of the responses, as patients may believe it is more acceptable to have forgotten a dose than to have intentionally/purposely not taken it, i.e., social desirability bias. And if non-adherent patients refuse to participate because they consider their behaviors unacceptable, this will skew prevalence estimates for those behaviors downwards [52, 95–97]. At the same time, self-report helps gain a deeper insight into how IS is taken (i.e., number of pills taken per dose, doses taken) and why (i.e., open question on adherence) [96, 98]. Because our analyses of patients’ behaviors rely quite heavily on those patients’ underlying intentions, we assume our findings offer a firm basis for future research on targeted interventions [46, 96].

Although our operational definition implied a link between non-persistence and rational decision making, we did not approach non-persistence as INA. This sample’s IS non-persistence rate (i.e., discontinuation of the regimen) was very low (*N* = 7, 0.5%). This finding echoed those of other studies, all of which reported very small prevalence (0.6%–3.1%) of medication non-persistence [17, 18, 49]. In all cases, including cases with a high relative rate of missing information on INA—e.g., Spain, Italy, Germany—, the number of cases involved were too low to allow in-depth analyses. Still, considering the clinical impact of non-persistence; [20, 65, 99, 100], further insight is needed to determine, for example, whether this measurement arises from a misunderstanding of the question. For example, there needs to be a clear distinction between interruptions in IS use that arise from regimen changes versus those where, contrary to their clinicians’ advice, patients simply abandon their IS regimens for prolonged periods; [101–103]. The former represents a therapeutic adjustment, the latter a potentially life-threatening behavior based on a conscious but misguided (and hopefully preventable) decision [67, 83].

Also, as this was a cross-sectional study, no longitudinal data were collected. Therefore, it is not possible to draw inferences regarding INA’s development or evolution. Patients were asked about their non-adherence over the last month. This cannot cover possible life-cycles of INA behaviors (i.e., it is not possible to say whether patients go through phases during which the type and level of non-adherence behaviors change) [92, 104]. While current findings suggest that non-adherence increases over time, [52, 57, 66, 70], applying these findings to INA will require data on intentionality and negative perceptions (worries) collected across multiple time points. In short, capturing INA’s dynamic underlying nature will require further longitudinal research [105].

### Conclusion

Based on a validated measurement (i.e., the BAASIS^©^) of intentional non-adherence to immunosuppressive medication (INA) [32], and referring to Fishbein’s Integrative Model of Behavioral Prediction to further understand INA-relevant behavior, this large multi-center study assessed the prevalence of INA on an international level. INA occurs when patients intentionally alter their medication regimens against medical advice, i.e., via drug holidays and/or dose alteration. Our analyses indicated that the correlates most strongly associated with INA were having a university-level education, belonging to an ethnic minority, or lacking health insurance that covered IS costs. As reasons, patients commonly cite worries (e.g., burdensome side-effects) or barriers (e.g., constraints related to their medication regimens), or a desire to regain a sense of control over their lives. In addition to highlighting the importance of patient-level factors associated specifically with INA, these findings support the development and use of individually-tailored interventions to decrease INA.

## Data Availability

The raw data supporting the conclusion of this article will be made available by the authors, without undue reservation.

## Abbreviations

HTx, Heart transplantation; INA, Intentional nonadherence; IS, Immunosuppressive medication.
